# Intense Testing and Use of Vitamin D Supplements Leads to Slow Improvement in Vitamin D Adequacy Rates: A Cross-Sectional Analysis of Real-World Data

**DOI:** 10.3390/nu16010111

**Published:** 2023-12-28

**Authors:** Rodis D. Paparodis, Dimitra Bantouna, Evangelos Karvounis, Ioannis Zoupas, Sarantis Livadas, Nicholas Angelopoulos, Shahnawaz Imam, Dimitrios T. Papadimitriou, Juan C. Jaume

**Affiliations:** 1 Endocrinology, Diabetes and Metabolism Clinics, Private Practice, 26221 Patras, Greece; 2 Center for Diabetes and Endocrine Research, College of Medicine and Life Sciences, University of Toledo, Toledo, OH 43614, USA; shahnawaz.imam@utoledo.edu; 3 Hellenic Endocrine Network, 10682 Athens, Greece; dimitra.bantouna@yahoo.gr (D.B.); sarntis@gmail.com (S.L.); drangelnick@gmail.com (N.A.); info@pedoendo.net (D.T.P.); 4 Department of Medicine, Edward Hines, Jr. VA Hospital, Loyola University Chicago, Hines, IL 60141, USA; juan.jaume@va.gov; 5 Endocrine Surgery Center of Excellence, Euroclinic Hospital, 11528 Athens, Greece; karvounis@endocrinesurgeon.gr; 6 School of Medicine, University of Athens, 11527 Athens, Greece; gianniszp@outlook.com.gr; 7 Division of Endocrinology, Diabetes and Metabolism, Athens Medical Center, 11528 Athens, Greece; 8 Endocrinology, Diabetes and Metabolism Clinics, Private Practice, 11528 Athens, Greece; 9 Endocrinology, Diabetes and Metabolism Clinics, Private Practice, 65302 Kavala, Greece; 10 Medical School, University of Thessaly, 41223 Larisa, Greece

**Keywords:** vitamin D deficiency, vitamin D supplementation, vitamin D insufficiency, vitamin D adequacy, cholecalciferol

## Abstract

Background: Vitamin D testing (VDT) and supplement use (VDS) are on the rise, but most patients remain deficient (<30 ng/mL-VDD). We designed the present real-world study to assess this paradox. Methods: We reviewed data from all patients visiting our clinics between 2014 and 2022. We estimated the rate of patients with vitamin D adequacy (≥30 ng/mL) (VDA) by year and month of testing, the dose of VDS (low (≤1200 IU/day), medium (1201–3000 I/day) and high dose (>3000 IU/day)), intake duration (short-term (<12 months) and long-term use (≥12 months)), and timing of use (current use, former use, no use). Results: We enrolled n = 6912 subjects with vitamin D measurements: n = 5195 females (75.2%), age 44.0 ± 16.8 years, BMI 27.9 ± 6.5 kg/m^2^; never users: n = 5553 (80.3%), former users: n = 533 (7.7%), current users: n = 826 (12.0%). Current use of VDS was higher in females. VDT rose from 42.1% in 2014 to 92.7% in 2022, and VDA rose from 14.8% to 25.5% for the same time. VDA was found overall in n = 1511 (21.9%); Never users: n = 864 (15.6%), Former users: n = 123 (23.2%); and Current users: n = 370 (44.8%). The maximal VDA (67.9%) was found in subjects using high-dose VDS in the long term. Conclusions: Despite the significant rise in VDT and VDS use, VDA was found in a minority of patients. Prolonged use of high-dose supplements produces modest improvements in VDA.

## 1. Introduction

Vitamin D is a steroid hormone produced by the skin and hydroxylated to its main form (25-OH-cholecalciferol) in the liver [1]. Although vitamin D is converted to the active hormone calcitriol (1–25 di-hydroxyvitamin D) after a second hydroxylation in the kidney, which binds to the nuclear vitamin D receptor (VDR), there is evidence to suggest that vitamin D itself has its own health benefits [1]. The presence of inadequate concentrations of vitamin D is known as vitamin D deficiency (VDD) and is one of the most puzzling entities of modern medicine, characterized even as “The big Vitamin D mistake” [2], referring to the erroneous statistical calculations regarding the estimation of the recommended dietary allowance for vitamin D [3]. Scientific meetings focusing on vitamin D research have become sites of lively debate due to the presentation of conflicting data on the subject. The single most challenging issue in vitamin D research is the definition of VDD [4]. Different organizations use different serum concentration cutoffs, with decent rationales supporting each view. Most scholars agree that vitamin D concentrations lower than 20 ng/mL constitute some degree of VDD. Some believe that lower cutoffs (e.g., 15 or 12 or 10 ng/mL) are even more appropriate [5], while others are in favor of the original 2011 Endocrine Society Clinical Practice Guideline, which supports the implementation of 30 ng/mL as the cutoff of sufficiency [6]. Independent of each clinician’s or researcher’s view, VDD remains an extremely hot topic for both physicians (resulting in 38,189 results on a PubMed search on 8 March 2023) and the general public alike (more than 219 million results on Google search the same day). Guidelines for vitamin D supplementation or replacement strategies have proposed different regimens, including doses that range from 400 IU to 10,000 IU daily, depending on the perspective and patient-centered treatment goals [6,7,8,9,10].

Despite the availability of multiple such Clinical Practice Guidelines and the wide availability of vitamin D supplements in our country, erratic treatment strategies have been observed, either due to the treating clinician, the poorly compliant patient, or the self-medication of multiple patients, given the over-the-counter nature of some vitamin D supplements. In our clinical practice, we have noted a steady increase in serum vitamin D measurements lately, along with a rising trend for use of vitamin D supplements in our population. Given this trend, we would expect a substantial improvement in the vitamin D concentrations in a large number of patients. Instead, vitamin D sufficiency has rarely been found, leading to disappointment and mistrust in patients and physicians alike regarding the efficacy of the medical treatments available. Since many patients have been offered somewhat lower doses and/or have been treated for short periods of time, we decided to assess whether these factors were the leading causes for our clinical observations. Therefore, we designed the present study to investigate this controversy.

## 2. Materials and Methods

The endocrinology, diabetes, and metabolism clinics in Patras, Greece, serve as a regional referral center for physicians of all specialties in Western Greece, including all clinical scenarios involving the entire spectrum of endocrine and metabolic disorders. In this population, which is representative of the general population of the Western Greece region, we have enrolled all willing subjects in a prospectively collected and daily updated patient registry, where clinical, laboratory, diagnostic, and imaging data, the medical therapies or interventions, and their outcomes are documented. Our registry includes patients’ data since 1 March 2014. For the present study, we gathered and analyzed data collected during the first visit of our subjects to our clinics, from patients enrolled in our registry from March 2014 up to December 2022. The data analyzed consist of clinical information, such as gender, menopausal status, age, height and weight, blood pressure, heart rate, history of vitamin D supplement (VDS) use, their dose and duration of treatment, date of cessation of VDS intake, and the serum 25(OH)D concentration on study enrollment, when available, along with the year and month of blood testing. The body mass index (BMI) was estimated using the following equation: BMI = Weight (kg)/Height (m)^2^. VDS in Greece are sold over the counter and on prescription, and consist of cholecalciferol (vitamin D3).

Subjects who were using VDS consistently on study enrollment underwent a measurement of serum vitamin D concentration, if none was available. Our labs measure vitamin D concentration with a competitive radioimmunoassay (range 5.8–100.0 ng/mL, sensitivity 1.9 ng/mL), which correlates strongly with the gold standard measurement with liquid chromatography–mass spectroscopy (DiaSource ImmunoAssays^®^, Louvain-la-Neuve, Belgium) [11]. Some measurements before December 2015 were performed with other equivalent, commercially available radioimmunoassays [12].

### 2.1. Inclusion Criteria

We enrolled all subjects who signed informed consent to participate in the registry, provided adequate clinical information, and had a measurement of serum 25(OH)D within 2 months from registry enrollment. It is worth noting that our population is largely homogeneous racially (Caucasians only) and environmentally (geographic region).

### 2.2. Exclusion Criteria

We excluded subjects with a history of unclear use of vitamin D supplements, or when vitamin D measurement was not available.

### 2.3. Subjects Grouping

For methodological reasons, we categorized our subjects into three groups, based on their history of prior use of vitamin D supplements during the study enrollment interview: never users (N), if there was no history of intake of any vitamin D-containing supplement, ever; former users (F), if they had consistently taken any vitamin D-containing supplement for at least 2 consecutive months, but had stopped doing so any time prior to study enrollment; current users (C), if they had been consistently taking any vitamin D-containing supplement for at least 2 consecutive months and were still doing so at study enrollment. It is important to note that no foods are fortified with vitamin D in Greece as of yet (July 2023), but vitamin D preparations are available both as over-the-counter supplements and as prescription medications, which can be commonly and freely prescribed by physicians of all specialties. 

Prespecified subgroups of the C and F subjects, based on the mean daily dose used, included the following: C subjects who are/were taking low-dose vitamin D supplements (daily average ≤ 1200 IU), those who are/were taking moderate doses of vitamin D supplements (daily average 1201–3000 IU), and those who are/were taking high-dose vitamin D supplements (daily average > 3000 IU).

Prespecified subgroups of the C and F patient groups included the following subgroups as well: subjects who had been taking vitamin D supplements for ≤12 months—short-term users (ST), and those who had been taking vitamin D supplements for >12 months—long-term users (LT).

### 2.4. Statistical Analysis

Statistical analysis and figure generation were performed using GraphPad Prism v.5.0 (GraphPad Software^®^, Boston, MA, USA) and MedCalc Statistical Software version 22.007 (MedCalc^®^ Software Ltd., Ostend, Belgium). The normality of the distribution of nominal data was assessed using the Kolmogorov–Smirnov test. When the data did not follow the normal distribution, we log-transformed them and assessed them for normality using the Kolmogorov–Smirnov test again. We compared means with Student’s *t* test or 1-way ANOVA, or the non-parametric Kruskal–Wallis test, when the data did not follow the normal distribution. A correction for multiple comparisons was made using Bonferroni’s multiple comparison test for data following the Gaussian distribution and Dunn’s multiple comparison test for data not following the Gaussian distribution. For categorical variables, we compared proportions using Fischer’s exact test or chi-squared (χ^2^) test, when >2 variables were analyzed. *p* values < 0.05 were deemed statistically significant.

A multivariate logistic regression model was constructed in order to identify factors that are likely to independently affect the possibility of achieving vitamin D adequacy, including the following parameters: age, gender, BMI, status of vitamin D supplement use (F vs. C vs. N), dose (low vs. high), and duration of use (short term vs. long term).

### 2.5. Ethical Considerations

All subjects signed informed consent to participate in the present study. The Institutional Review Board’s approval was not obtained, since this is not required by Greek laws for non-interventional studies in private research institutions.

## 3. Results

**Figure 1:**
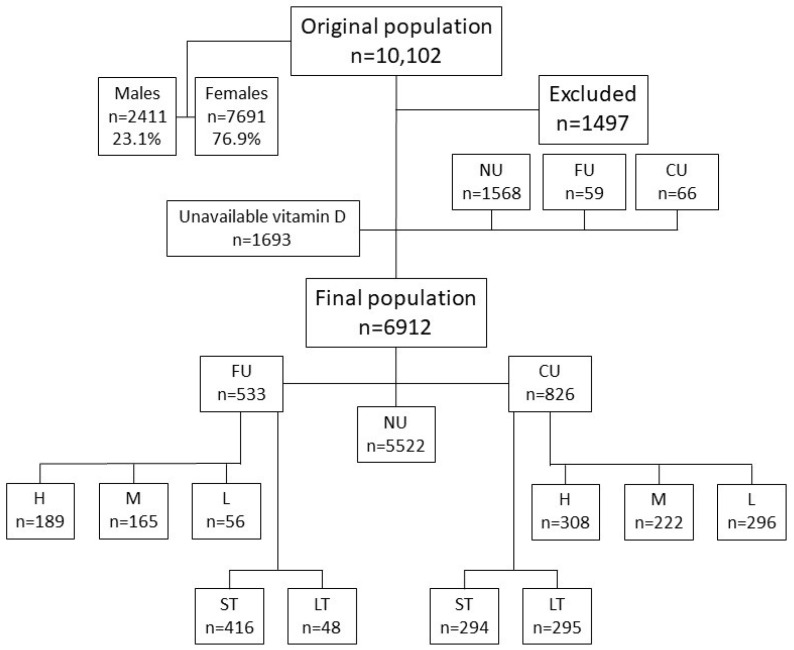
A flow diagram of the study enrollment. Legend: NU: never users of vitamin D-containing supplements; FU: former users of vitamin D-containing supplements; CU: current users of vitamin D-containing supplements. H: high dose of vitamin D-containing supplements (>3000 IU daily); M: medium dose of vitamin D-containing supplements (1200–3000 IU daily); L: low dose of vitamin D-containing supplements (≤1200 IU daily); ST: short-term use of vitamin D-containing supplements (<12 months); LT: long-term use of vitamin D-containing supplements (≥12 months).

We reviewed the charts of 10,102 consecutive patients attending our clinics between 1 March 2014 and 31 December 2022. After applying exclusion criteria, n = 6912 subjects participated in the present study, the majority (n = 5195, 75.2%) being females. A flow diagram of the study enrollment is shown in Figure 1. The baseline characteristics of the entire cohort and each major subjects’ subgroup are presented in Table 1. The number of subjects in each group, subgrouped by the dose of VDS used in each gender, is presented in Table 2. It is of note that the use of VDS was statistically significantly less common in males compared to females (*p* < 0.001). Males used VDS much less frequently compared to females overall. Their percentage ranged from 9.8 to 16.2% in the former and current users, which are significantly lower compared to the never users’ (N) group: 27.8% (*p* < 0.001, Table 2).

Overall, n = 1718 women were in menopause (33.1%), while n = 3477 were in their fertile years (66.9%). The women in menopause were more likely to be C (OR 3.53, 95% CI 2.98–4.17, *p* < 0.001) but not F users (OR 1.12, 95% CI 0.92–1.36, *p* = 0.30) of VDS (C: 391/1718 (22.8%), F: 169/1718 (9.8%), and N: 1158/1718 (67.4%)) compared to younger women (C: 268/3477 (7.7%), F: 308/3477 (8.9%), and N: 2901/3477 (83.4%)). 

The prevalence of vitamin D inadequacy (<30 ng/mL) in the entire cohort was 76.9% (5319/6912) and was lower in C users of VDS (55.2% (456/826)) compared to F users of VDS (76.8% (409/533)) or N users of VDS (84.4% (4689/5533)), with *p* < 0.001 for all comparisons.

### 3.1. Monthly Distribution

**Figure 2:**
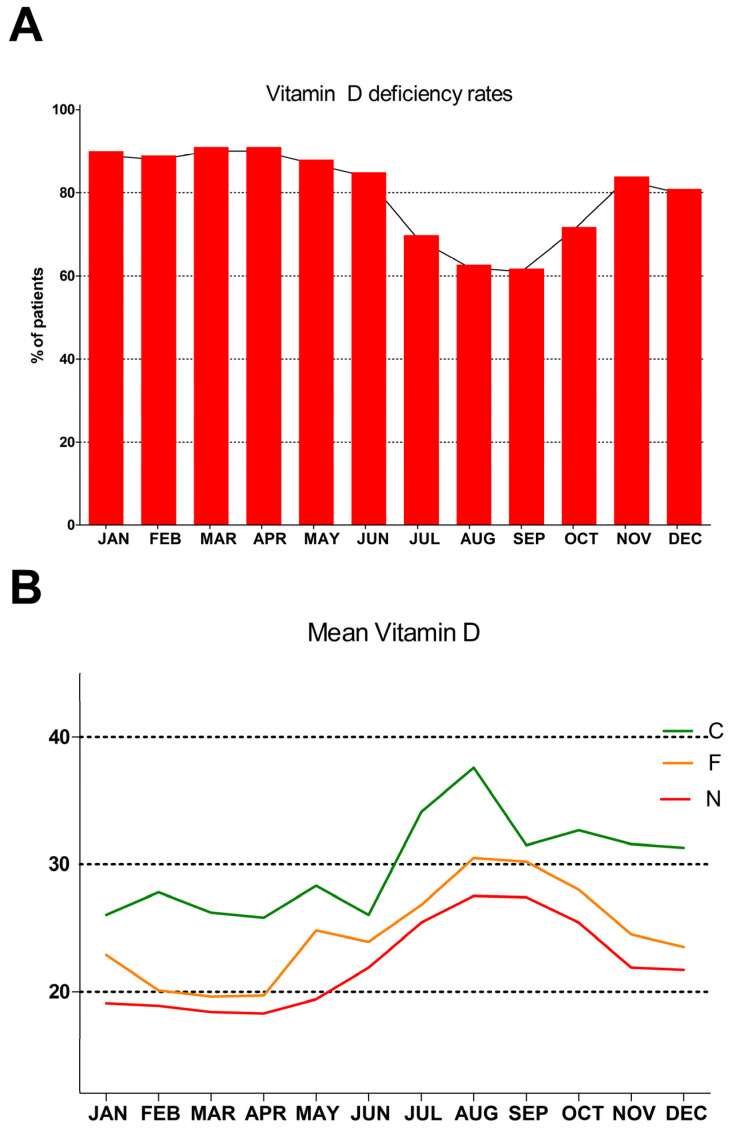
(**A**) The monthly rate of vitamin D deficiency (<30 ng/mL) of the entire cohort. (**B**) The monthly mean serum vitamin D concentration of each subgroup split by the history of vitamin D supplement use: never users (N) (those who never used any vitamin D-containing supplement), former users (F) (those who used vitamin D-containing supplements in the past), and current users (C) (those who currently use vitamin D-containing supplements).

The monthly distribution of mean serum vitamin D concentrations of the entire cohort and each subgroup, along with their comparisons, are presented separately in Table 3 and depicted in Figure 2. It is worth noting that in both N and F subjects, the mean serum vitamin D concentration never reaches adequacy (>30 ng/mL), while in the current users’ subgroup (C), it is found within the adequacy range between July and December. The rates of VDA of the entire population by month are presented in Table 3. The majority of the study subjects remained below the adequacy range all year long.

### 3.2. Vitamin D across the Years

**Figure 3:**
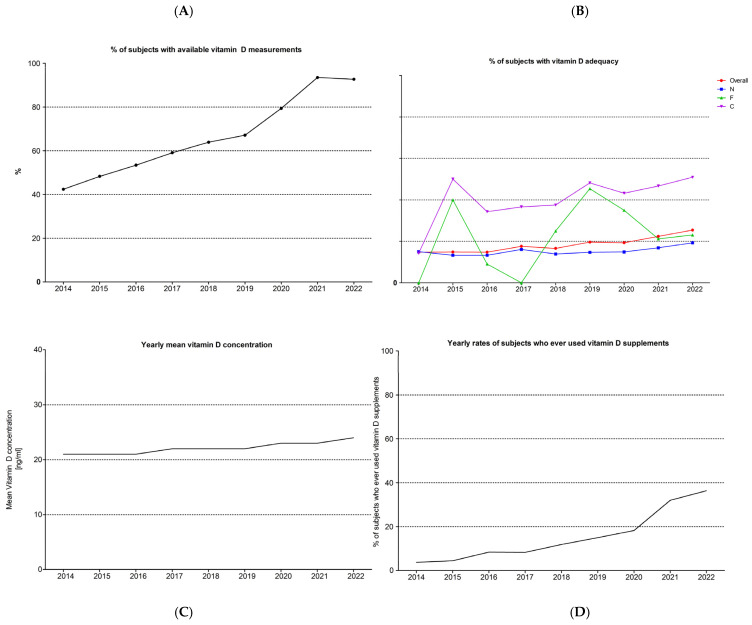
(**A**) The frequency (%) of available vitamin D measurements in the entire clinic’s patient population each year. (**B**) The rate (%) of subjects with vitamin D adequacy (>30 ng/mL) per year. (**C**) The mean serum vitamin D concentration (ng/mL) of the entire cohort per year of study. (**D**) The rate (%) of subjects with any history of vitamin D-containing supplements use per year.

Vitamin D testing was available in 68.1% of all patients visiting our clinics (6912/10,102), and this rate increased significantly over the years from 42.4% in 2014 to 92.7% in 2022. The actual numbers for each year are presented in Table 4 and depicted in Figure 3A. The yearly number of subjects in each subgroup and the rate of vitamin D adequacy are presented in Table 5 and depicted in Figure 3B. The mean concentration of vitamin D in the entire population each year is presented in Table 5 and depicted in Figure 3C. The number of subjects ever using VDS is presented in Table 5 and depicted in Figure 3D. Despite the rising VDT and the drop in never users’ rate, the improvement in vitamin D adequacy is low, reaching only 25.5% in 2022.

Since this small rise in the mean vitamin D concentration could be potentially attributed to the rise in the use of vitamin D supplements, we estimated the mean vitamin D concentration of the never users (N) each year. These means are presented in Table 5 and depicted in Supplemental Figure S1. Overall, a small but statistically significant change was observed (1-way ANOVA, *p* value 0.0043), with statistically significantly higher mean serum vitamin D concentrations observed in 2022 compared to the respective values of the years 2015 and 2016 only.

### 3.3. Supplementation Dose and Duration Effects

The effects of the duration of VDS related to the dose used are presented in Table 6 and Supplemental Figure S1. Overall, out of 1359 subjects who had ever used VDS (C or F groups), n = 520 (37.2%) had vitamin D concentrations > 30 ng/mL. Long-term treatment with VDS (>12 months) resulted in statistically significantly higher rates of VDA (>30 ng/mL), especially in subjects taking high doses continuously (*p* < 0.0001). Even in that population, though, VDA was achieved only by 68%. In all subgroups, current use of VDS resulted in higher rates of VDA compared to prior use. 

Our subjects never exposed to vitamin D supplements had a mean 25-OH-D concentration of 21.9 ± 9.0 ng/mL, while n = 864/5553 (15.6%) had vitamin D adequacy (>30 ng/mL). This concentration was statistically significantly lower compared to all subjects’ subgroups who are currently using vitamin D supplements (current users), independent of the dose used (low dose n = 296, mean 25-OH-D = 28.0 ± 10.1 ng/mL; medium dose n = 222, mean 25-OH-D = 29.3 ± 10.6 ng/mL; high dose n = 308, mean 25-OH-D = 31.3 ± 13.3 ng/mL, *p* < 0.05 compared to the never users’ group for all subgroups). Similar findings were present when the mean vitamin D concentrations were compared to those of former vitamin D users, who had taken medium- or high-dose supplements (high dose n = 246, mean 25-OH-D = 25.2 ± 8.5 ng/mL, and medium dose n = 214, mean 25-OH-D = 24.2 ± 8.6 ng/mL, *p* < 0.05 compared to the never users’ group for both subgroups), but not those who had used low-dose vitamin D supplements in the past (n = 73, mean 25-OH-D = 24.3 ± 7.6 ng/mL, *p* > 0.05 compared to the never users’ subgroup). The rates of vitamin D adequacy (>30 ng/mL) were higher compared to the never users in all subgroups of current users (high dose n = 148/308 (48.1%), medium dose n = 104/22 (46.8%), low dose n = 118/296 (39.9%)) and former users of vitamin D supplements (high dose n = 66/246 (27.0%), medium dose n = 45/214 (21.2%), and low dose n = 12/73 (16.4%)); *p* < 0.05 for all comparisons. Similarly, the rates of vitamin D adequacy were higher in all subgroups of current users of vitamin D supplements compared to all subgroups of former users of vitamin D supplements (*p* < 0.05 for all comparisons).

### 3.4. Multivariate Regression Analysis

The output of the multivariate regression analysis model is presented in Table 7. The model was highly statistically significant, revealing positive associations of vitamin D adequacy (>30 ng/mL) and the current use of VDS (strongest correlation), male gender, and age, while negative correlations were found between vitamin D adequacy and BMI and low-dose supplement use.

## 4. Discussion

Vitamin D sufficiency and deficiency remain highly controversial definitions in the global medical literature, mostly due to the lack of agreement on a “normal” concentration for the general population [13,14]. While there continues to be debate as to what the definition of vitamin D deficiency is, the circulating concentration should be at least 30 ng/mL according to the Endocrine Society’s guidelines, which is still valid [6]. In addition, doses of 50,000 IU weekly (or 6–7000 IU daily) were deemed necessary to achieve adequacy (>20 ng/mL or 50 nmol/L) in most patients deficient in vitamin D, while the elderly and obese would require up to 2–2.5 times more [6]. Nevertheless, some studies assessing the efficacy of medications employed to treat osteoporosis required subjects to receive low-dose supplementation (400 IU daily) [15] οr loading doses of 50,000–60,000 IU [16]. All these studies (and many more) were limited by the failure to see vitamin D as a hormone, for which supplementation would aim to achieve a specific blood concentration or to normalize some parameters of calcium metabolism, i.e., lowering–normalizing PTH [17], similar to what is seen with levothyroxine supplementation with regard to serum TSH normalization. Studies such as the VITAL study [18], which was the largest to-date multicentric, prospective, randomized, placebo-controlled trial, attempted to supplement their subjects with 2000 IU of cholecalciferol daily, despite their population being largely sufficient in vitamin D (average vitamin D concentration 31 ng/mL). Therefore, it came as no surprise that such a huge, well-designed, and well-executed protocol found no obvious clinical benefit from the intervention compared to placebo. Furthermore, it is well known that 2000 IU daily of cholecalciferol is far from the 6000 IU daily that would be required from all sources to ensure that 97.5% of the population reaches and maintains a 25(OH)D concentration of 30 ng/mL [2].

In the present study, we present our findings from an eight-year continuous observation of a real-world population originating from a general endocrinology practice in the region of Western Greece. It is important to note that Western Greece is a region with less than average sunshine hours per year in the overall sunny country of Greece [19], rendering it an area at a somewhat higher risk for vitamin D deficiency compared to the rest of the country. In our practice, vitamin D sufficiency was found extremely rarely overall, and even more so in patients not using supplements. This finding is consistent with previous observations in the Greek population [20,21], Europe [9,22], and globally [23], but this was the first time that patients were stratified according to the use of supplements, their dose, and the duration of use. It is worth noting that vitamin D deficiency persists in our population all year long, despite significant improvements during the late summer and early fall months. These monthly changes in serum vitamin D concentrations are related to the effects of increased exposure to UVB radiation [24] and are expected, given the small duration of vitamin D winters and the large amounts of UVB radiation delivered to the Greek population between April and September each year [25]. Indeed, based on the findings reported by a randomized controlled trial from Ireland in patients with Crohn’s disease, exposure to sunshine has been found to enhance the effects of vitamin D supplements in raising serum vitamin D concentrations [26]. Severe VDD has been described in the elderly citizens of Athens, Greece, in the past [21], but our study is the first to delve into the use of VDS in women according to their menopause status in Greece. In that regard, we present a significantly higher likelihood of use of such agents in postmenopausal women compared to their younger counterparts, most likely as part of osteoporosis or osteopenia treatment strategies. Similar to our findings, common use of VDS in postmenopausal women has been found to produce higher concentrations of serum 25(OH)D in a recent study of 319 healthy women from Slovenia as well [27]. It is worth noting that VDA (>30 ng/mL) is achieved only in about half of our patients who use supplements continuously, a finding consistent with what is commonly described in clinical trials of vitamin D supplementation, when personalized treatment strategies, based on the baseline vitamin D concentration of each patient alone, were not implemented [28]. 

The most significant finding in the present study is that vitamin D sufficiency is low in all subgroups of patients, whether on or off vitamin D supplements, and it only goes up to 67% when vitamin D supplements are used continuously for >12 months in mean daily doses > 3000 IU. Lower doses, or smaller duration of use, decrease the efficacy of these strategies, resulting in sufficiency rates < 50%, especially when small doses are used for short periods of time (e.g., use of mean daily doses ≤ 1200 IU for <12 months leads to sufficiency in 28.4% of our subjects). This is consistent with the results of a recent meta-analysis, which highlighted that a longer duration of therapy or higher amounts of vitamin D supplements increase the likelihood of achieving vitamin D sufficiency in postmenopausal women [27]. The commonly used on/off strategies, where patients are placed on therapy for some months, only to resume therapy a few months or years later, appear completely ineffective in maintaining the vitamin D concentrations within the reference range. This effect is independent of the duration of VDS intake, or the dose employed, given that the maximal rate of VDA achieved is 35%, when high doses (mean daily dose > 3000 IU) have been used for >12 consecutive months but the patients discontinue treatment. This rate drops to 18.4%, similar to what is seen in the untreated group, when smaller doses (≤1200 IU daily) are used for shorter periods of time. This is well highlighted in other studies as well, where a long-term maintenance dose seems indispensable in order to prevent vitamin D concentrations from dropping below normal values [29].

A second very important point is that we have observed a steep rise in VDT in recent years, where almost all patients undergoing blood tests for any reason tend to include vitamin D measurements in their panel. This is consistent with findings from various studies, including a large cohort study from Liverpool, UK, where the claims for VDT rose 11-fold between 2007 and 2012 [30]. Similarly, the Ireland’s Health Service Executive (HSE) Primary Care Reimbursement Services (PCRS) pharmacy claims database identified a strong increase in the use of vitamin D supplements in women aged > 55 years, and those diagnosed with breast cancer between 2005 and 2011, even though the median dose remained stable, at an average of 857 IU daily [31]. This could also be related to the well-established rise of supplement use during the COVID-19 pandemic [32], likely in response to some studies yielding favorable results in the outcome of infection from this virus, when vitamin D supplements are used, even though not all studies and meta-analyses agree on that [33,34,35]. Despite that explosive rise in VDT, the rise in VDS use is much slower and the rates of adequacy achieved are even much lower, mostly limited to those using high-dose supplements continuously. Recently, an interesting novel public health strategy has been proposed regarding vitamin D repletion and supplementation, using the upper tolerable doses—as defined by the Endocrine Society’s guidelines’ expert committee—for 6–8 weeks, followed by continuous maintenance doses all year long except sunny vacations, which are characterized as not requiring medical supervision at Endocrine Society’s vitamin D guidelines and are at the same time recognized as upper tolerable by the former Institute of Medicine (IOM) [36,37]. Our results are in complete accordance with such a strategy that seems able to assure vitamin D sufficiency in the optimal range of 40–60 ng/mL, minimizing costs for testing and medical supervision. Moreover, in our series, among those with continuous daily supplementation with doses >3000 IU, there were no incidents of vitamin D intoxication or hypersensitivity, with no subject exceeding 100 ng/mL and the upper safety limit being 150 ng/mL [38]. 

All these observations suggest that the treatment of vitamin D deficiency seems to be more a matter of individualized care, which requires personalization, rather than “one size fits all” treatment strategies [39]. Based on the information obtained from our study, we feel that an alternative tailor-made clinical approach may be needed, where the treatment protocol would factor in the following parameters: a. the vitamin D concentration desired, b. the patient’s baseline calcium metabolism including assessment of serum concentration of vitamin D together with that of PTH, c. the presence or absence of factors, which could alter vitamin D dynamics, such as malabsorptive vitamin disorders, poor calcium intake, etc., and d. obesity, which is a risk factor for vitamin D deficiency, especially given that the mean BMI of our population was in the overweight range, similar to what is observed in the Greek population [40], and e. genetic information pertinent to each individual, such as enzymes involved in vitamin D metabolism and genetic loci associated with specific risks for vitamin D deficiency and its outcomes, once such data become more widely available and clinically usable [41,42,43,44]. Also, planning vitamin D measurements could possibly enhance the ability to achieve and maintain sufficiency in more patients by increasing compliance, even though this remains to be proven by future research.

Our study is limited by the relatively small number of patients on current and past treatment, and especially the fact that the time since cessation of vitamin D supplements intake occurred. Also, its cross-sectional design does not allow for long-term observation data on the study population, even though most prior studies in the Mediterranean region described similar rates of vitamin D inadequacy in their populations. In addition, we are limited by the use of a population that is racially and environmentally homogeneous, rendering our results representative of similar populations globally. Additionally, our work did not include parameters related to genetic and epigenetic factors, which could play a role in the complex interplay of calcium and vitamin D metabolism and affect both the baseline vitamin D concentrations and those observed after specific treatments. Another noteworthy limitation of the present work consists of the use of patients evaluated at a specialty clinic, allowing some degree of selection bias regarding the subjects’ gender (since substantially more women are seen in our clinics), the indication for the clinic visit, the past medical history, and the concurrent medical therapies used. It is important to mention, though, that specialty care is widely and easily available for patients in Greece, who usually self-refer themselves with small or no medical complaints. Therefore, our study has a chance to assess the vitamin D supplement use patterns of a population assimilating the general population of the region. Despite the clear results presented in this study regarding vitamin D adequacy in our population, we suspect that our findings on the prevalence of vitamin D deficiency might be an underestimation because vitamin D measurement was performed in all consenting subjects who were taking vitamin D supplements, while it was not done so for those who never used vitamin D supplements. It is also important to note that the type of supplements used by our subjects, the correct route of administration, and the exact compliance with their intake were not assessed, potentially biasing our results. Finally, this work does not assess the indications or reasons for vitamin D measurement or supplementation in our population, which could produce some bias, since patients with endocrine disorders requiring lifelong treatment with vitamin D should be more consistent in their VDS intake compared to patients testing vitamin D for less clinically sound indications. Our main strengths consist of the inclusion of a large number of untreated patients, and of consecutive patients over 8 years in the analysis, which assimilates the real-world medical practice, as well as the identification of the differential effects of different regimens and different durations of treatment on vitamin D concentration. 

## 5. Conclusions

Overall, these real-world data raise concerns about the true status of vitamin D adequacy policies in the entire Greek and Southern European regions. Based on this work, future studies should aim to assess the effectiveness of strategies employed to achieve and maintain vitamin D adequacy in patients in need in order to measure clinical outcomes associated with the supplementation and treatment strategies employed.

## Figures and Tables

**Table 1:** Baseline characteristics of the entire cohort and each subgroup, split by mean serum vitamin D or the history of vitamin D supplement use.

	Units	Overall	D < 30	D > 30	N	F	C	*p* Value
n (%)	-	6912 (100)	5319 (76.9)	1593 (23.1)	5522 (80.3)	533 (7.7)	826 (12.0)	NA
Age	Years	44.0 (16.8)	44.2 (16.7) ^c^	47.5 (17.3)	45.7 ^a,b^ (16.4)	43.4 (15.9)	53.1 (16.0)	<0.0001
Heart rate	bpm	79.0 (12.1)	80.2 ^c^ (12.3)	77.8 (12.2)	79.0 (12.1)	78.3 (11.7)	78.3 (12.0)	0.85
Systolic BP	mmHg	125.0 (35.5)	125.5 (16.1)	124.8 (14.7)	124.8 ^b^ (27.7)	125.2 (14.0)	126.3 (16.9)	0.24
Diastolic BP	mmHg	78.5 (10.5)	78.5 ^c^ (10.1)	77.5 (10.0)	78.4 (10.6)	78.1 (9.4)	79.5 (9.2)	0.12
Height	meters	1.67 (0.08)	1.70 (0.05)	1.67 (0.10)	1.66 ^b^ (0.09)	1.64 (0.08)	1.63 (0.08)	<0.0001
Weight	kg	77.1 (25.6)	78.5 ^c^ (20.9)	73.4 (75.6)	77.7 ^b^ (26.5)	73.7 (17.3)	71.8 (16.4)	0.0002
Body mass index	kg/m^2^	27.9 (6.5)	28.2 ^c^ (6.7)	26.7 (5.8%)	28.2 (9.6)	27.4 (6.0)	27.0 (6.2)	0.09
Female gender n (%)	-	5195 (75.2)	4146 ^c^ (78.0)	1049 (65.9)	4009 (82.1) ^a,b^	478 (89.7)	708 (85.7)	<0.0001
Male gender n (%)	-	1717 (24.8)	1173 ^c^ (22.0)	544 (34.1)	1544 (27.9) ^a,b^	55 (10.3)	118 (14.3)	

Legend: All measures are presented as means (SD), unless otherwise noted. SD: standard deviation; NA: non-applicable; N: never users of vitamin D supplements; F: former users of vitamin D supplements; C: current users of vitamin D supplements; ^a^ statistically significantly different compared to the F subgroup; ^b^ statistically significantly different compared to the C subgroup; ^c^ statistically significantly different compared to the >30 ng/mL subgroup.

**Table 2:** Number of subjects who have used vitamin D supplements in the past or are currently using them, split by gender, and the percentage of males in each subgroup. χ^2^ test *p* < 0.001.

	CH	CM	CL	FH	FM	FL
Females	260	186	262	221	193	64
Males	48	36	34	25	21	9
%Males	15.6	16.2	11.5	10.2	9.8	12.3
Total	308	222	296	246	214	73

Legend: FH: former user of high-dose vitamin D supplements; FM: former user of medium-dose vitamin D supplements; FL: former user of low-dose vitamin D supplements; CH: current user of high-dose vitamin D supplements; CM: current user of medium-dose vitamin D supplements; CL: current user of low-dose vitamin D supplements.

**Table 3:** Mean vitamin D concentrations in the entire cohort and each subgroup, stratified by month of measurement, along with the rate of adequacy of vitamin D (>30 ng/mL).

Vitamin D	Overall	N	F	C	* p * Values
Mean ± SD >30/total %>30 ng/mL >20/total %>20 ng/mL	23.0 ± 9.6 1358/6912 19.6 4121/6912 59.6	21.9 ± 9.0 ^a,b^ 864/5553 15.6 3089/5553 55.6	24.7 ± 8.5 ^b^ 123/533 23.2 385/533 72.2	29.6 ± 11.6 370/826 44.8 647/826 78.4	<0.0001
JAN	20.2 ± 8.3 61/564 10.8 265/564 47.0	19.1 ± 7.4 ^a,b^ 34/456 7.4 194/456 42.5	22.9 ± 7.1 9/47 19.4 31/47 66.0	26.0 ± 11.8 17/60 28.3 40/60 66.6	<0.0001
FEB	20.1 ± 9.7 69/600 11.5 272/600 45.3	18.9 ± 9.5 ^b^ 38/487 7.7 194/487 39.8	20.1 ± 5.7 ^b^ 3/35 7.4 17/35 48.6	27.8 ± 9.4 29/78 36.7 61/78 78.2	<0.0001
MAR	19.4 ± 8.5 64/672 9.5 294/672 43.8	18.4 ± 7.6 ^b^ 35/541 6.5 215/541 39.7	19.6 ± 7.5 ^b^ 4/55 7.1 24/55 43.6	26.2 ± 11.7 25/77 32.2 55/77 71.4	<0.0001
APR	19.2 ± 8.8 51/508 10.0 216/508 42.5	18.3 ± 7.6 ^b^ 29/416 6.9 157/416 37.7	19.7 ± 7.5 ^b^ 3/39 6.7 19/39 48.7	25.8 ± 8.9 19/53 36.6 40/53 75.5	<0.0001
MAY	21.0 ± 8.1 77/608 12.6 297/608 48.8	19.4 ± 7.3 ^a,b^ 38/487 7.7 206/487 42.3	24.8 ± 7.2 8/36 21.4 28/36 77.8	28.3 ± 12.4 31/84 36.9 63/84 75.0	<0.0001
JUN	22.5 ± 8.2 101/632 16.0 361/632 57.1	21.9 ± 7.7 ^b^ 66/522 12.7 300/522 57.5	23.9 ± 8.7 8/29 27.3 20/29 69.0	26.0 ± 10.1 29/82 34.9 41/62 66.1	0.0051
JUL	26.7 ± 9.5 183/595 30.8 452/595 76.0	25.4 ± 8.9 ^b^ 121/463 26.1 332/463 71.7	26.8 ± 8.4 ^b^ 16/57 27.3 47/57 82.4	34.1 ± 10.6 47/75 62.1 73/75 97.3	<0.0001
AUG	28.7 ± 10.8 125/330 37.8 280/330 84.8	27.5 ± 9.6 ^b^ 90/259 34.7 213/259 82.2	30.5 ± 8.5 17/43 39.4 40/43 93.0	37.6 ± 18.0 18/29 63.6 27/29 93.1	0.0203
SEP	28.1 ± 10.0 248/639 38.8 519/639 81.2	27.4 ± 9.9 ^b^ 182/509 35.7 400/509 78.6	30.2 ± 8.7 29/55 52.4 50/55 90.9	31.5 ± 10.5 38/75 50.0 69/75 92.0	0.0026
OCT	26.5 ± 9.1 157/542 29.0 415/542 76.6	25.4 ± 8.7 ^b^ 104/434 24.0 324/434 74.7	28.0 ± 5.8 12/36 32.1 32/36 88.9	32.7 ± 10.5 42/71 58.2 59/71 83.1	<0.0001
NOV	23.2 ± 9.1 114/676 16.9 422/676 62.4	21.9 ± 7.8 ^b^ 65/548 11.8 323/548 58.9	24.5 ± 9.1 ^b^ 10/52 20.0 38/52 73.1	31.6 ± 12.8 39/75 51.7 61/75 81.3	<0.0001
DEC	23.0 ± 9.2 108/546 19.8 328/546 60.1	21.7 ± 8.7 ^b^ 64/430 14.8 231/430 53.7	23.5 ± 5.9 ^b^ 6/49 13.2 39/49 79.6	31.3 ± 10.3 38/66 56.9 58/66 87.9	<0.0001
* p * values	<0.0001	<0.0001	<0.0001	<0.0001	.

Legend: Vitamin D measured in ng/mL. SD: standard deviation. In the vertical column, comparisons are made between the monthly mean vitamin D concentrations of the N, F, or C subgroups. In the final horizontal column, comparisons are made between the monthly vitamin D concentration means within each group. N: never users of vitamin D supplements; F: former users of vitamin D supplements; C: current users of vitamin D supplements—see text for definitions; ^a^ statistically significantly different compared to the F group; ^b^ statistically significantly different compared to the C group.

**Table 4:** Number of vitamin D measurements presented to our clinics compared to the number of new patients examined each year in our clinics.

	2014	2015	2016	2017	2018	2019	2020	2021	2022
Tests	316	673	606	694	656	582	672	1474	1240
Patients	745	1395	1134	1174	1027	868	846	1576	1337
%Tests	42.4	48.3	53.4	59.1	63.9	67.1	79.4	93.5	92.7

Legend: Tests: number of new patients visiting our clinics with available vitamin D measurement each year; Total: number of new patients visiting our clinics each year; %Tests: percentage of the patients visiting our clinics with available vitamin D measurements each year.

**Table 5:** Yearly distribution of our subjects in the different subgroups, along with the percentage of those never exposed to vitamin D supplements (%N), and the rates of vitamin D adequacy (%>30).

	2014	2015	2016	2017	2018	2019	2020	2021	2022
Current users	9	23	45	53	73	73	96	239	214
Former users	3	6	5	4	5	14	26	233	237
Never users	304	643	555	637	578	495	550	1002	789
Total	316	673	606	694	656	582	672	1474	1240
%N	96.3	95.6	91.6	91.8	88.1	85.0	81.8	68.0	63.6
>30	47/316	100/673	90/606	122/694	109/656	114/582	130/672	330/1474	316/1240
%>30	14.8	14.9	14.8	17.6	16.6	19.6	19.3	22.4	25.5
Mean ± SD	22.0 ± 9.8	21.5 ± 8.7	21.5 ± 9.2	22.1 ± 9.1	22.0 ± 9.1	22.8 ± 10.5	23.2 ± 9.5	23.8 ± 9.4 ^b,c,d,e^	24.9 ± 10.3 ^a,b,c,d,e,f,g^
N mean ± SD	21.9 ± 9.8	21.2 ± 8.1	21.1 ± 8.9	21.6 ± 8.9	21.4 ± 8.3	21.7 ± 10.0	21.9 ± 8.6	22.4 ± 8.8	23.2 ± 9.5 ^b,c^

Legend: Vitamin D is measured in ng/mL; %N: percentage of our subjects never exposed to vitamin D supplements; >30: number of subjects with vitamin D measurement ≥ 30 ng/mL, divided by the total number of subjects enrolled in the study in the same year; %>30: percentage of our subjects achieving vitamin D adequacy (≥30 ng/mL) each year; Mean: mean vitamin D concentration in ng/mL; SD: standard deviation; N mean ± SD: mean vitamin D measurement of the never users’ subgroups ± their group-specific standard deviation; ^a^ statistically significantly higher than 2014; ^b^ statistically significantly higher than 2015; ^c^ statistically significantly higher than 2016; ^d^ statistically significantly higher than 2017; ^e^ statistically significantly higher than 2018; ^f^ statistically significantly higher than 2019; ^g^ statistically significantly higher than 2020.

**Table 6:** Vitamin D adequacy rates in each subgroup, taking into consideration the user status (C vs. F), the dose of vitamin D supplements used (H vs. L), and the duration of treatment (ST vs. LT).

	<30	>30	Total	%>30
CHLT	31	66	97	68.0
CHST	93	97	190	51.1
CMLT	57	71	128	55.5
CMST	60	46	106	43.4
CLLT	109	80	189	42.3
CLST	84	33	117	28.2
FHLT	13	7	20	35.0
FHST	187	64	251	25.5
FMLT	12	4	16	25.0
FMST	131	39	170	22.9
FLLT	15	3	18	16.7
FLST	47	10	57	17.5
Total	839	520	1359	37.2

Legend: CHLT: current user of high-dose vitamin D supplements for >12 months; CHST: current user of high-dose vitamin D supplements for ≤12 months; CMLT: current user of medium-dose vitamin D supplements for >12 months; CMST: current user of medium-dose vitamin D supplements for ≤12 months; CLLT: current user of low-dose vitamin D supplements for >12 months; CLST: current user of low-dose vitamin D supplements for ≤12 months; FHLT: former user of high-dose vitamin D supplements for >12 months; FHST: former user of high-dose vitamin D supplements for ≤12 months; FMLT: former user of medium-dose vitamin D supplements for >12 months; FMST: former user of medium-dose vitamin D supplements for ≤12 months; FLLT: former user of low-dose vitamin D supplements for >12 months; FLST: former user of low-dose vitamin D supplements for ≤12 months.

**Table 7:** Multifactorial regression analysis of the association between several clinical parameters and the likelihood of achieving vitamin D adequacy (VDA). Coefficient B in bold signifies statistically significant associations.

	Coefficient B	Standard Error	z-Value	*p* Value	Odds Ratio	95% Conf. Interval
Male gender	**0.33**	0.08	4.27	<0.001	1.4	1.2–1.63
Age	**0.01**	0.00	4.44	<0.001	1.01	1.01–1.01
Low-dose supplement	−**0.61**	0.18	3.33	<0.001	0.55	0.38–0.78
High-dose supplement	0.18	0.15	1.19	0.23	1.2	0.89–1.63
Former use	0.78	0.8	0.98	0.33	2.19	0.46–10.42
Current use	**1.66**	0.8	2.08	0.04	5.27	1.1–25.3
Long-term treatment	0.25	0.8	0.32	0.75	1.29	0.27–6.23
Short-term treatment	−0.54	0.79	0.68	0.5	0.58	0.12–2.75
BMI	−**0.05**	0.01	8.30	<0.001	0.95	0.94–0.96
Constant	−0.63	0.17	3.72	<0.001	0.53	.

## Data Availability

The raw data of the present study are available upon reasonable request.

## Supplementary Materials

The following supporting information can be downloaded at: https://www.mdpi.com/article/10.3390/nu16010111/s1, Figure S1: The effects of the duration of VDS related to the dose used.
