# Supplementary material for: Intense Testing and Use of Vitamin D Supplements Leads to Slow Improvement in Vitamin D Adequacy Rates: A Cross-Sectional Analysis of Real-World Data
Source: Nutrients. 2023 Dec 28;16(1):111. doi: 10.3390/nu16010111 (PMC10780961; doi:10.3390/nu16010111)
Supplement: Supplementary file 1 [file nutrients-16-00111-s001.zip › nutrients-2736236-supplementary.pdf]

Supplementary Materials

Intense Testing and Use of Vitamin D Supplements Leads to Slow Improvement in Vitamin D Adequacy Rates: A Cross-Sectional Analysis of Real-World Data

Rodis D. Paparodis, Dimitra Bantouna, Evangelos Karvounis, Ioannis Zoupas, Sarantis Livadas, Nicholas Angelopoulos, Shahnawaz Imam, Dimitrios T. Papadimitriou and Juan C. Jaume

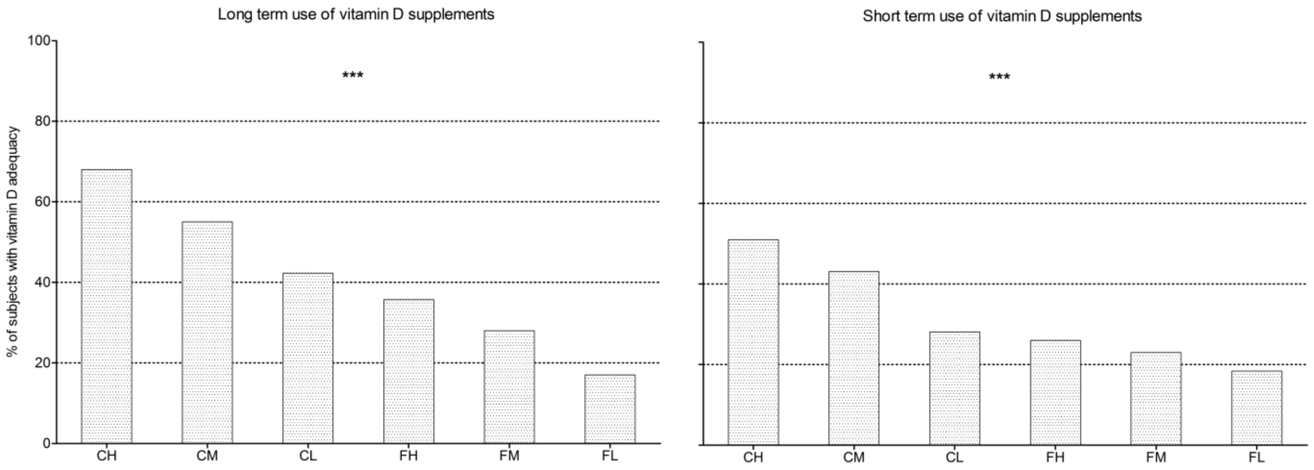

Figure S1. The effects of the duration of VDS related to the dose used. A. Subjects with long term use of vitamin D containing supplements. B Subjects with short term use of vitamin D containing supplements. Legend: ***: p<0.001.
